# Supplementary material for: Effects of Prolonged Pomace Contact on Color and Mouthfeel Characteristics in Merlot Wine During the Ageing Process Under Microwave Irradiation
Source: Foods. 2025 Feb 5;14(3):507. doi: 10.3390/foods14030507 (PMC11816411; doi:10.3390/foods14030507)
Supplement: Supplementary file 1 [file foods-14-00507-s001.zip › foods-3416176-supplementary.pdf]

Table S1. Criteria for the sensory evaluation of wine

Index	Evaluation criteria	Score
Alcohol	No strong alcohol flavor	8-10
	Slight alcohol taste	5-7
	Stimulating alcohol odor	0-4
Colour	Red brown, bright colour	8-10
	Orange yellow, relatively bright colour	5-7
	Rust red, dull colour	0-4
Clarity	Uniform liquid with high clarity	8-10
	Relatively uniform with high clarity	5-7
	Relatively turbidity and non-uniform liquid	0-4
Bitterness	No perception of bitterness	8-10
	Slight bitterness but acceptable	5-7
	Feel some bitterness	0-4
Astringency	No astringency, sweet aftertaste	8-10
	Slightly astringent, weak sweet taste	5-7
	High astringency, weak aftertaste	0-4
Persistence	Smooth, no stimulate the mouth	8-10
	Light acidity, taste sour	5-7
	High acidity, weak persistence	0-4
mouthfeel	Refreshing smooth, non irritation to the mouth	8-10
	Slightly acidity, slightly stimulation to the mouth	5-7
	High acidity, significant irritation to the mouth	0-4
Quality	Excellent quality, highly recommendation	8-10
	Good quality, recommendation	5-7
	Acceptable, not recommendation	0-4
